# OncoAlert Round Table Discussions: The Global COVID-19 Experience

**DOI:** 10.1200/GO.20.00603

**Published:** 2021-04-06

**Authors:** Gilberto Morgan, Evandro de Azambuja, Kevin Punie, Felipe Ades, Kathrin Heinrich, Nicola Personeni, Ramy Rahme, Roberto Ferrara, Kevin Pels, Marina Garassino, Michael von Bergwelt-Baildon, Gilberto Lopes, Fabrice Barlesi, Toni K. Choueiri, Howard Burris, Solange Peters

**Affiliations:** ^1^ Department of Medical Oncology, Skåne University Hospital, Lund, Sweden; ^2^ Medical Oncology Clinic, Institute Jules Bordet, l'Université Libre de Bruxelles (U.L.B), Brussels, Belgium; ^3^ Department of General Medical Oncology, University Hospitals Leuven, Leuven, Belgium; ^4^ Centro de Oncologia, São Paulo, Brazil; ^5^ Department of Medicine III, University Hospital, LMU Munich, München, Germany; ^6^ Department of Biomedical Sciences, Humanitas University, Milan, Italy; ^7^ Medical Oncology and Hematology Unit, Humanitas Clinical and Research Center—IRCCS, Milan, Italy; ^8^ Hôpital Saint Louis, Université Paris Diderot, Paris, France; ^9^ Department of Medical Oncology, Thoracic Oncology Unit, Fondazione IRCSS, Istituto Nazionale dei Tumori Milano, Milan, Italy; ^10^ Dana-Farber Cancer Institute, Harvard Medical School, Boston, MA; ^11^ Division of Medical Oncology, Department of Medicine, Sylvester Comprehensive Cancer Center at the University of Miami, Miami, FL; ^12^ Gustave Roussy Cancer Center, Villejuif, France; ^13^ Sarah Cannon Research Institute, Tennessee Oncology, Nashville, TN; ^14^ Service d'oncologie médicale, CHUV, Lausanne, Switzerland

## Abstract

The speed and spread of the COVID-19 pandemic has been affecting the entire world for the past several months. *OncoAlert* is a social media network made up of more than 140 oncology stakeholders: oncologists (medical, radiation, and surgical), oncology nurses, and patient advocates who share the mission of fighting cancer by means of education and dissemination of information. As a response to the COVID-19 pandemic, OncoAlert hosted *The Round Table Discussions.* We have documented this effort along with further discussion about the COVID-19 pandemic and the consequences on patients living with cancer to disseminate this information to our colleagues worldwide.

## INTRODUCTION

**FIG 1:**
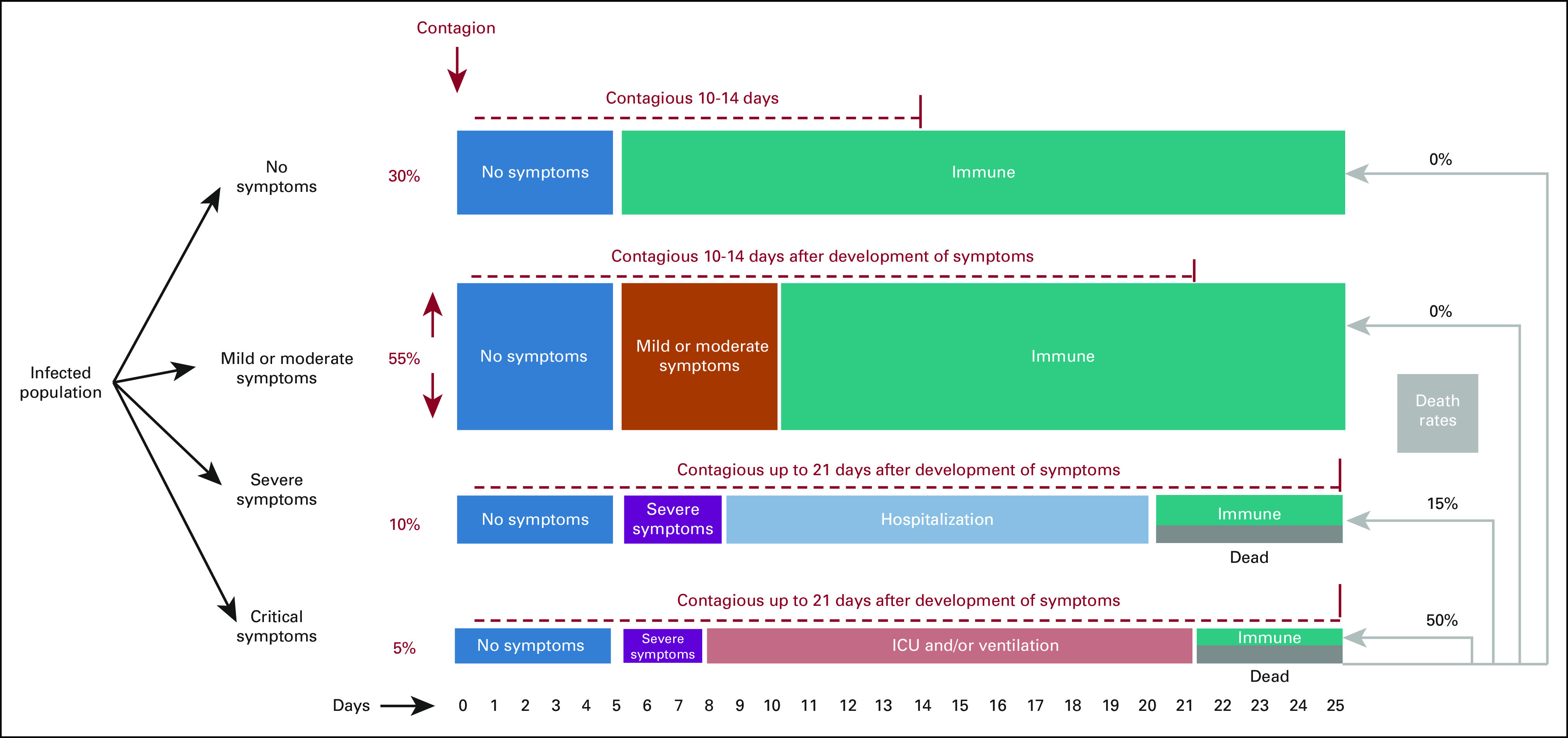
Timeline of infection.^37-40^

On December 31, 2019, an outbreak of pneumonia of unknown cause in Wuhan City, China, was reported to the WHO. On January 10, 2020, the WHO issued a travel advisory, and on January 30, the agency declared the novel coronavirus outbreak a public health emergency of international concern. Although epidemiologists and infectious disease researchers were still seeking to understand its transmission and mortality (Fig. 1), COVID-19 spread quickly to the rest of the world.^1,2^ Although mortality rates have varied by country, patients living with cancer were quickly identified as a vulnerable population.

**CONTEXT**

**Key Objective**
Is it possible for medical social media to contribute in global emergencies, such as the COVID-19 pandemic?
**Knowledge Generated**
The OncoAlert network is a social media network made up of more than 140 oncology stakeholders. Through social media initiatives like the round table discussions, the OncoAlert network was able to disseminate information on the treatment of COVID and useful logistical advice on how to deal with the pandemic. This was done at a time when little was known about the pandemic or the effects it had on patients with cancer. The initiatives of the network also addressed resilience and aimed at helping colleagues cope with the stress that comes with being a healthcare professional during the pandemic.
**Relevance**
Oncology social media can have a positive impact in times of emergency. The COVID-19 pandemic has shown us that a reliable network can play a role in filtering and amplifying important information for all stakeholders.

### The Early Stages of the Outbreak and Tracing

Strict social distancing, travel restrictions, and contact tracing are interventions that were implemented to halt the spread within China.^3^ Following the Chinese example, many governments instituted nationwide lockdowns. Conversely, South Korea adopted alternative strategies based on mass testing and contact tracing of symptomatic patients with epidemiologic links.^4^ Although contact tracing is a successful strategy in the early stages of an outbreak, its logistics become more challenging as the number of contacts increases.^5^

### Prevention and Control

Two measures to control the spread of coronavirus have been universally accepted: social distancing and hygiene measures, especially hand washing.^6^

Different degrees of social distancing have been used with success in China, Italy, Switzerland, and Spain to reduce the spread of COVID-19.^7-12^

The formulation of public health policies is context sensitive and multifactorial, depending on the previous knowledge of the disease, population social and health habits, income, and health education.^13^ As a consequence, during the first wave, European countries affected later had more time to gather this information and decrease the rate of infection. However, implementation has varied depending on the degree to which public authorities made decisions based on scientific data.^12,13^ In facing the pandemic, governments have had to deal with consequences, not only in public health but also social, political, and economic. Although certain governments have adapted stricter approaches and declared national emergencies, others adopted a more flexible approach with prime focus on respecting core societal values, placing trust on public responsibility.^14^ The response from societies has been mixed; however, one thing that has become increasingly clear as more developing countries face the pandemic is the clear economic and political inequalities in global policy responses exposing the world's most vulnerable.^15^

At the beginning of the pandemic, the use of masks was neither mandatory nor recommended.^16^ We now understand that although surgical and handmade tissue masks do not protect the wearer from infectious respiratory droplets, they do decrease the likelihood of transmission by reducing the spread of such droplets.^16-18^ For COVID-19, the WHO recommends surgical masks in low-risk situations and respirators for high-risk situations and this has led to masking being adopted and recommended in several countries as a public policy.^19,20^

The basic reproduction number (R_0_) is an indicator of the contagiousness and transmissibility of infectious agents. It is one of the fundamental and most often used metrics for the study of infectious disease dynamics. Dietz states that R_0_ is the number of secondary cases that one case would produce in a completely susceptible population.^21^ Estimations of the R_0_ value are calculated as a function of three primary parameters: (1) the likelihood of infection per contact between a susceptible person and an infectious person or vector (transmissibility), (2) the contact rate, and (3) the duration of contagiousness after a person becomes infected,^21^ 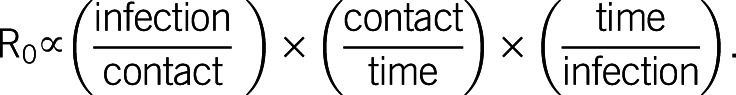


The R_0_ estimation has important implications for future pharmaceutical and nonpharmaceutical interventions. If the R_0_ value is 2.2, the minimum threshold for hypothetical herd immunity needed for disease extinction is 55% (ie, > 55% of the population must be immune, through either vaccination or previous infection, to achieve herd immunity). However, if the R_0_ value is 5.7, the threshold rises to 82%.^22^

Now that we are at the peak of the second wave and our hospitals are once again at capacity, there is dire need for good prevention and control. However, as people tire from the situation and receive the news of a US Food and Drug Administration (FDA)–approved vaccine, they are also becoming more relaxed about travel, social distancing, hand washing, and mask wearing. This in combination with the holiday season is a deadly combination that promises to be an extra burden on our healthcare system.

### Keeping Healthcare Workers Healthy and On Duty

Healthcare workers (HCW) are vulnerable to COVID-19 because of the high risk of contagion from presymptomatic and asymptomatic subjects,^23^ as well as the aerosol or surface stability^24^ and high transmissibility^25^ of the SARS-CoV-2 virus, particularly in the hospital environment. Data from China's National Health Commission showed that since February 2020, more than 3,300 HCW have been infected and 22 have died.^26^ The global picture is even bleaker: for example, in Italy by April 2020, 13,121 HCW had been infected and more than 142 doctors and 30 nurses died.^27^ As the pandemic accelerates, preventing intrahospital infections and transmission is a key concern.^28^ In healthcare settings, providing masks and other personal protective equipment such as gloves or face shields is essential for both worker and patient safety, but shortages have been reported in many facilities.^29^ Many countries have recommended and have implemented constant screening and testing for workers who have been in close contact with infected people. Considering that symptoms may underestimate positive cases^30^ and that transmission may occur presymptomatically,^31^ SARS-CoV-2 tests should ideally be offered to medical personnel and routinely repeated to keep the hospital environment safe; however, not every country is following the same implementation. Segregated team workflow with designated wards for management of suspected cases among patients and personnel^32^ and use of telemedicine (outpatient consultations online or by phone) are other crucial measures that must be universally implemented to minimize risk to patients and staff and provide a continuum in cancer treatments at the same time.^33^ However, one thing we have seen is that the standards and the infrastructure for telemedicine vary quite dramatically by geographic region.

### The Mental Stress on the Oncology Task Force

Physical workload, uncertainties, and anxiety increase mental stress for HCW during the pandemic.^34^ Increased workloads are compounded by the necessity of quarantine for infected coworkers, resulting in longer hours and extra on-call duties.^35^ For oncology professionals, the vulnerability of patients to COVID-19 because of both their cancer status and risk of adverse outcomes on therapy increases that stress. Oncologists are expected to make tremendously difficult decisions on whom to provide with intensive care treatment while weighing prognosis and comorbidities.^36^ Meanwhile, explaining these decisions to patients has become harder; an end-of-life discussion in full protective gear without the presence of family makes a difficult task more unpleasant. Additionally, the risk and fear of bringing the infection home is always present, with some colleagues deciding to distance themselves from their family to protect them; others establish strict decontamination regimens when returning from work to ensure their families’ safety. HCW with children also must balance practical arrangements because of school and childcare closures. Coping methods and resilience can mitigate the mental stress on short- and long-term well-being and will be key elements in burnout prevention (Table 1).

To examine the stress the COVID-19 pandemic has put on HCW, the ESMO resilience task force in collaboration with the OncoAlert network put out two surveys, one in April, 2020, titled “Impact of COVID-19 in oncology professionals” and the other released in July titled “The current Normal.” These surveys are intended to better understand the impact that all these factors associated with the pandemic had on the daily life of HCW and how this could possibly lead to stress and burnout.^41^

### Oncologists Treating Patients With COVID-19

Oncologists worldwide are involved in the care of SARS-CoV-2–infected patients living with cancer. Patients with COVID-19 are presenting with abnormal findings from their blood smear such as lymphopenia and cytokine storm. This requires experience in diagnosis and management, a task for which oncologists are cross-qualified because of their experience treating older patients with complex infections or side effects of certain immune therapies. Furthermore, oncology departments have significant volume and resources and experience in clinical research. However, oncologists simultaneously treat patients without COVID-19 in different parts of the hospital or in some cases, different buildings.

**TABLE 1:**
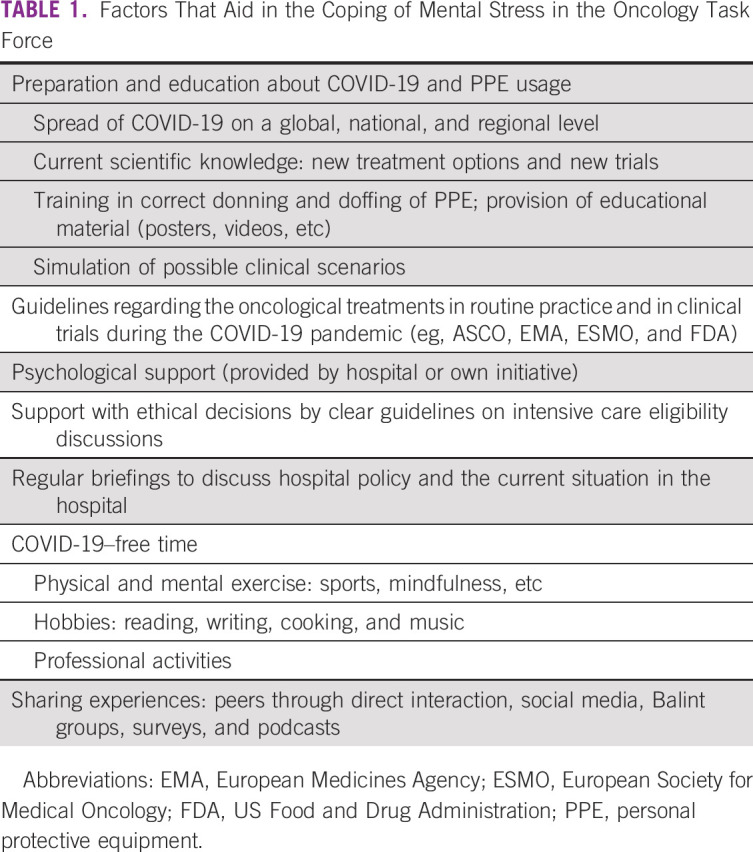
Factors That Aid in the Coping of Mental Stress in the Oncology Task Force

**TABLE 2:**
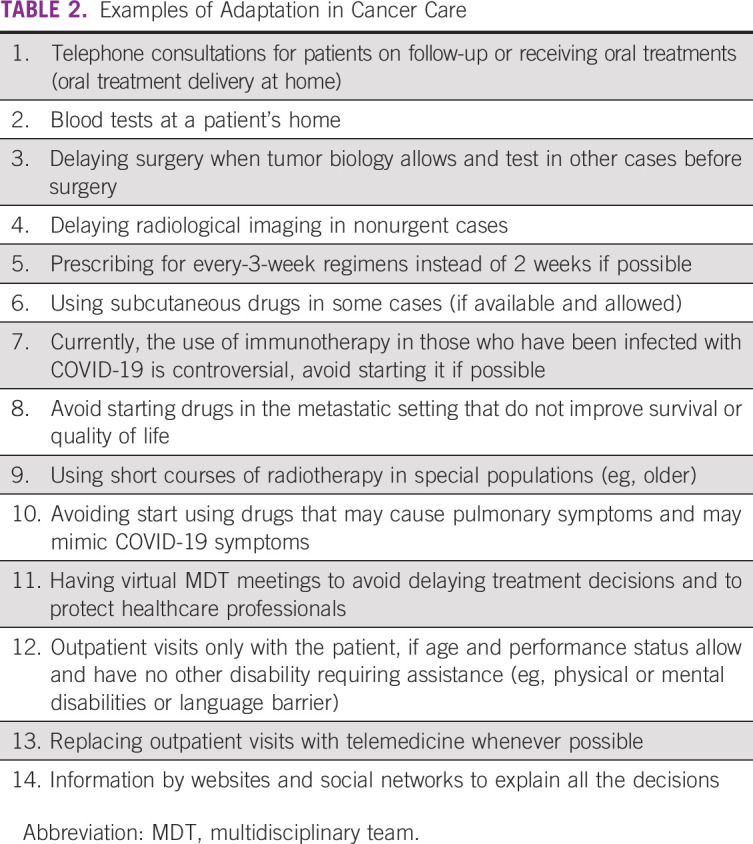
Examples of Adaptation in Cancer Care

### The Effects of the COVID-19 Pandemic on Patients With Cancer

In the beginning of the pandemic, patients with cancer were labeled as high risk for COVID-19, especially those under treatment as they were thought to be at an increased risk of mortality. This label was not without consequences, as it led to changes in cancer management, decreasing the doses of therapies, modification of immunotherapies, and switching from intravenous to oral chemotherapies in many patients. Two small studies reporting COVID-19 outcomes in those with cancer concluded that patients with cancer are more susceptible to contracting COVID and also have a risk of developing symptoms with higher severity. Other studies determined that severity was depending on the type of cancer, those with hematological malignancies having a greater risk of having more severe complications from COVID-19.^42^ These patients in general required more intensive supportive interventions and had an increased risk of death. As the pandemic went on, studies showed receiving chemotherapy within 4 weeks of a positive COVID-19 test does not contribute to a more severe disease or a predictor of death from the virus. Similar studies observed the same for hormonal therapies, targeted therapies, radiation therapies, and immunotherapies.^43^ This allowed oncologists to be less apprehensive about continuing therapy in patients with cancer during the pandemic and to give a treatment that is not compromised by changes.

However, it is not just those with a current cancer diagnosis who are affected. The extent of the damage will have a toll on future cancer diagnosis because of stopping or reduction of cancer screening and deferment of routine diagnostics. There is a projected increase in the number of preventable cancer deaths as a result of delays because of the pandemic. In a study based in the United Kingdom (UK), it has been estimated that these delays will hit hardest among four different tumor groups: breast, colorectal, esophageal, and lung cancers with more than 3,600 avoidable deaths in the United Kingdom alone.^44^ To deal with the effects of the pandemic on cancer, we will need to increase routine diagnostic capacity or public health messaging and make urgent policy changes to deal with the backlog within diagnostics.^44^

On December 11, 2020, the US FDA issued an emergency authorization to the first COVID-19 vaccine.^45^ The introduction of the vaccine will no doubt bring about changes to patients with cancer. The current question is who will get the vaccine, as patients with cancer were not included in the COVID-19 vaccine clinical trials, yet are a population at risk. Another important thing to consider is the effect of societal inequities, as Black patients with new cancer are more susceptible for COVID-19 infections.^46^ Because of the limited supply of the vaccine and financial resources, it could take several months before they reach the communities where those who are most susceptible live.

### Adapting Cancer Care During the COVID-19 Pandemic

During the COVID-19 pandemic, professional societies such as American Society of Clinical Oncology (ASCO) and European Society for Medical Oncology (ESMO) are making recommendations for modifying care of different tumor types.^47^

Deviations from standard practice must always be discussed with patients so that they continue to feel that they are partners in treatment decisions. Specialized nurses may regularly contact patients to ensure their well-being. When pursuing curative treatment, delays in surgery or chemotherapy may be acceptable if tumor biology allows. If a standard treatment fails in the curative setting and a patient transitions into the palliative setting, treatment breaks may be considered if they do not prolong survival or improve the quality of life. Hospitalized patients face restrictions to visits; however, some countries have been more flexible with patients receiving end-of-life treatment, allowing them to receive limited visitors. There are many adaptions to cancer care during the pandemic (Table 2), and new practices should be incorporated as needed to improve patient outcomes.

Clinical trials have faced a huge suspension in screening and random assignment of new patients, and patients already on protocols have faced disruptions to how they are treated and followed. Although it has been possible to conduct some clinical trials, a variety of protocol deviations have had to be adopted. These deviations have to be aligned with the local standard for social containment and done to avoid risk of COVID-19 infection for the patient.^48^ Both the EMA (European Medicine Agency) and the US FDA have released guidance for sponsors on how to better accommodate patients in clinical trials during this pandemic.

### Challenges and Opportunities for the Scientific Community and Oncology Societies

This pandemic has had implications on academic oncology, and although the approval of an SARS-nCoV-2 vaccine seems to be coming soon, there is still great uncertainty over when or if we will again have meetings with tens of thousands of attendees. For that reason, oncology societies are moving toward a virtual experience in which everyone can enjoy the science without needing to travel or gather in large groups.

The first cancer society to take this on was the American Association for Cancer Research (AACR) offering 20 high priority presentations with free access to all during this virtual meeting. One immediate advantage was an increase in participants from 22,500 in 2019 to more than 61,000 in 2020. The second large virtual meeting came from ASCO, who delivered the entire scientific meeting, 2,300+ presentations, including 100+ on-demand broadcast sessions, with 42,750 registrants, including 40,000 oncology professionals, from 138 countries. Access was free to ASCO members, and there was a reduced rate for nonmembers.

The ESMO Congress in September was also 100% virtual and free for ESMO members. The Science weekend of ESMO20 boasted more than 30,000 registrants from more than 150 countries, and 2,137 abstracts were presented.

Both ASCO and ESMO provided a separate educational program on a different date, and both were 100% virtual.

The decision to implement virtual meetings comes with benefits such as improved global representation, decreased costs to institutions, lower emissions by reducing air travel, improved accessibility for the disabled, and allowing more patient advocates to attend.^49^ Videoconferencing has opened possibilities to simplify collaborations, which could also play a role during congresses as virtual meetings could happen in real time during the congress.

Another survey collaboration between ESMO and OncoAlert took place in July and examined the impact of COVID-19 as perceived by the oncology community. This survey was filled out by more than 1,000 participants, is projected for publishing in January 2021, and could give us new insights into telemedicine, virtual meetings, and how the pandemic has affected communication among oncology stakeholders on social media.

The OncoAlert network was started in 2019 with the purpose of sharing oncology news and practice changing information to colleagues worldwide in real time. Now, its members are normally more than 50% of the top social media influencers at every congress in which they participate, indicating the degree of dissemination of information and the growing number of colleagues who trust the OncoAlert network to keep them up to date. Although there is still room for development and greater cooperation with oncology societies, the COVID-19 pandemic has increased the network’s role in the sharing of emerging studies and data and in the case of these three surveys, determining how the pandemic has affected our colleagues.

## DISCUSSION

The COVID-19 pandemic has had a huge impact on cancer care worldwide. It created an urgent need not only to modify how treatments were delivered and patients were followed but also the ways that HCW interact with patients and each other. These changes have been burdensome physically, mentally, and emotionally. To address these increased burdens, some countries have created a psychological consultation hotline for HCW to help them cope. Hospitals have also created a direct support line for patients during this difficult period. With many meetings being canceled and/or transformed into a virtual format, the pandemic has also forced us to think of alternative ways of delivering and sharing scientific knowledge. In September during the ESMO Congress, the ESMO Resilience task force in collaboration with OncoAlert presented a proffered paper “The Impact of COVID-19 on the Oncology Professional” and the results were from two surveys. The results from the first survey (April) showed that 38% had feelings of burnout; this increased to 49% by the second survey (July). In April, 78% of participants felt an increased concern for their personal safety, and the proportion of professionals at risk of distress increased from 25% from the first survey to 33% by the second.^50^ One thing for certain is that the COVID-19 pandemic is affecting the well-being and performance of oncology professionals, and these issues must be addressed. The collaboration of the OncoAlert network with the ESMO resilience task force continues, with new surveys planned to better understand the impact of this pandemic in cancer care, the daily life of HCW, and stress or burnout.

Clinical trials will continue to face disruption during this pandemic; however, with time and guidance from agencies like the EMA and US FDA, disruptions will most likely be minimized. There will also be continuous financial implications to hospitals serving oncology patients, since many procedures (surgeries and/or imaging) have been canceled or delayed to protect our vulnerable patients. Once current conditions change, we must ensure that all patients will be able to return and be provided with proper care. However, some changes brought on by COVID-19 are here to stay, and the future will include an increased incorporation of telemedicine and virtual meetings.

In conclusion, although the global fight against the COVID-19 pandemic continues, we are seeing how some countries have been successful in flattening the curve and not overburdening their health systems. Although we hope that the rate of infection will decrease in response to further preventative measures and potentially with the mass implementation of a vaccine in early 2021, we also know that even if this happens, the effects of the second wave have already started to take their toll on the healthcare system and HCW. As the pandemic continues, oncologists, like many other specialists, are continuing to pay a high price in the form of an increased risk of infection, as well as mental and physical stress. The purpose of raising awareness of these issues is that the global healthcare community has to be better prepared to deal with this or any future pandemic and able to protect the HCW charged with doing so. Although the accelerated approval of a COVID-19 vaccine is a giant step in the right direction, it is very likely that a vaccine will not be administered to 75% of the global population before fall 2021. For this reason, we must have the right infrastructure, planning, testing capabilities, and willingness to act to decrease the amount of death from this pandemic. The OncoAlert network takes its responsibility to colleagues and patients very seriously and will be there every step of the way forming collaborations, educating, and reporting on COVID-19 for the duration of this pandemic and any other that may come.
